# *Vitis rotundifolia* Genes Introgressed with RUN1 and RPV1: Poor Recombination and Impact on *V. vinifera* Berry Transcriptome

**DOI:** 10.3390/plants13152095

**Published:** 2024-07-29

**Authors:** Mengyao Shi, Stefania Savoi, Gautier Sarah, Alexandre Soriano, Audrey Weber, Laurent Torregrosa, Charles Romieu

**Affiliations:** 1 UMR AGAP Institute, University Montpellier, CIRAD, INRAE, Institute Agro, 34090 Montpellier, France; mengyao.shi@supagro.fr (M.S.); gautier.sarah@inrae.fr (G.S.); alexandre.soriano@cirad.fr (A.S.); audrey.weber@inrae.fr (A.W.); 2 Department of Agricultural, Forest and Food Sciences, University of Turin, Largo Paolo Braccini 2, 10095 Grugliasco, TO, Italy; stefania.savoi@unito.it; 3 UMT Geno-Vigne^®^, IFV-INRAE-Institute Agro, 34060 Montpellier, France; laurent.torregrosa@institut-agro.fr; 4 LEPSE, University Montpellier, CIRAD, INRAE, Institute Agro, 34060 Montpellier, France

**Keywords:** hybrids, fruit development, *V. rotundifolia*, *V. vinifera*, transcriptome, resistance genes

## Abstract

Thanks to several *Vitis vinifera* backcrosses with an initial *V. vinifera* L. × *V. rotundifolia* (previously *Muscadinia rotundifolia*) interspecific cross, the MrRUN1/MrRPV1 locus (resistance to downy and powdery mildews) was introgressed in genotypes phenotypically close to *V. vinifera* varieties. To check the consequences of introgressing parts of the *V. rotundifolia* genome on gene expression during fruit development, we conducted a comparative RNA-seq study on single berries from different *V. vinifera* cultivars and *V. vinifera* × *V. rotundifolia* hybrids, including ‘G5’ and two derivative microvine lines, ‘MV102’ (resistant) and ‘MV32’ (susceptible) segregating for the MrRUN1/RPV1 locus. RNA-Seq profiles were analyzed on a comprehensive set of single berries from the end of the herbaceous plateau to the ripe stage. Pair-end reads were aligned both on *V. vinifera* PN40024.V4 reference genome, *V. rotundifolia* cv ‘Trayshed’ and cv ‘Carlos’, and to the few resistance genes from the original *V. rotundifolia* cv ‘52’ parent available at NCBI. Weighted Gene Co-expression Network Analysis (WGCNA) led to classifying the differentially expressed genes into 15 modules either preferentially correlated with resistance or berry phenology and composition. Resistance positively correlated transcripts predominantly mapped on the 4–5 Mb distal region of *V. rotundifolia* chromosome 12 beginning with the MrRUN1/MrRPV1 locus, while the negatively correlated ones mapped on the orthologous *V. vinifera* region, showing this large extremity of LG12 remained recalcitrant to internal recombination during the successive backcrosses. Some constitutively expressed *V. rotundifolia* genes were also observed at lower densities outside this region. Genes overexpressed in developing berries from resistant accessions, either introgressed from *V. rotundifolia* or triggered by these in the *vinifera* genome, spanned various functional groups, encompassing calcium signal transduction, hormone signaling, transcription factors, plant–pathogen-associated interactions, disease resistance proteins, ROS and phenylpropanoid biosynthesis. This transcriptomic insight provides a foundation for understanding the disease resistance inherent in these hybrid cultivars and suggests a constitutive expression of NIR NBS LRR triggering calcium signaling. Moreover, these results illustrate the magnitude of transcriptomic changes caused by the introgressed *V. rotundifolia* background in backcrossed hybrids, on a large number of functions largely exceeding the ones constitutively expressed in single resistant gene transformants.

## 1. Introduction

Grapevine (*Vitis vinifera* L.) holds significant agricultural importance as the major species cultivated worldwide within the *Vitaceae* family, with thousands of cultivars dedicated to wine grapes, table grapes, or raisins production [1]. Grapevine industries face significant challenges due to the prevalence of fungal diseases, particularly powdery and downy mildews, caused by the biotrophic pathogen *Erysiphe necator* and *Plasmopara viticola*, respectively [2]. Powdery mildews were thought to have originated in the eastern and central United States and were imported to Europe by the middle of the nineteenth century. Within a short period of time, they spread throughout Europe devastating all vineyards established with the susceptible Eurasian *vinifera* [3,4]. The massive use of fungicides required for their control become a major concern for European viticulture [5]. Grapevine breeding has a long history, with efforts to develop disease-resistant varieties dating back to the 19th century [4,6]. Interspecific hybridization aims to combine the high-quality fruit characteristics of the Eurasian *V. vinifera* with the disease resistances of wild *Vitis* species from other continents, including the more distant *V. rotundifolia* [6]. *V. rotundifolia*, also known as the muscadine grape, is native to the southeastern United States and has inherent resistance to many pests and diseases. Ongoing grapevine breeding programs now prioritize traits related to agronomy and production, such as yield, quality, and disease resistance. These characteristics are crucial in determining the acceptance of new grape varieties by viticulturists and the market value of the grapes [7]. Despite these advancements, resistant cultivars still require some level of plant protection, highlighting the complexity of breeding efforts and the ongoing need for innovation [7,8]. The enhancement of agronomic and production traits in grapes should be facilitated by a deeper understanding of their developmental and physiological characteristics. Over the past 25 years, numerous studies have been conducted to characterize grapevine fruit development at the molecular level. Transcriptomics was used to characterize the varietal diversity between a cultivated grapevine variety and the PN40024.V4 reference genome [9,10], as well as to understand the molecular response of grapevine berries to water deficit [11,12], temperature [13], combined stress [14], abscisic acid (ABA) [15] and pathogens [16], although these last ones are more frequently studied on leaves [14,17]. Comparative transcriptomic analysis between cultivated and wild species revealed the conservation of expressed genes [18]. Recently, a study explored the genetic diversity of nucleotide-binding leucine-rich repeat receptor (NLR) genes between wild and domesticated grapevine populations [19]. By analyzing 17 genotypes, they identified and classified these NLR genes into eight distinct types, discovering that wild populations generally possess a higher number of these genes compared to cultivated varieties. Additionally, gene ontology (GO) enrichment analysis indicated a reduction in programmed cell death-associated gene families, a key immune response, in domesticated grapevines. This suggests that domestication may have led to a decrease in the pool of resistance-related genes, as observed in tomatoes [20]. However, the practical consequences of such impairment in defense gene number should be interpreted with care, since biotic and abiotic selective pressures drastically change between vineyard and grapevine natural habitats [3,21].

Genetic improvement through hybridization, particularly incorporating fungus-tolerance traits, is a key focus for developing new grapevine cultivars [22,23]. The MrRUN1/MrRPV1 locus proved very convenient to confer a high level of tolerance to powdery and downy mildews [24,25,26,27,28]. MrRUN1/MrRPV1 were introgressed in *vinifera* according to a pseudo-backcrossing breeding scheme [29]. The ‘G5’, ‘G14’, and ‘Artaban’ genotypes, which were obtained after the fourth and fifth backcrosses of a *V. vinifera* × *M. rotundifolia* G52 hybrid with *V. vinifera* exhibit a high level of tolerance against downy and powdery mildews [7,26,27,30]. Screening of a BAC library from a powdery mildew-resistant plant allowed narrowing down the RUN1/RPV1 locus to a ~1 Mbp in chromosome 12 [30]. A cluster of 11 resistance gene analogs (RGAs) was identified within this locus including seven genes encoding full-length TIR-NBS-LRR resistance proteins. Sequencing of this region ruled out the presence of other kinds of resistance against fungal pathogens [30]. A novel approach for multi-domain and multi-gene family identification provided insights into the evolutionary dynamics of disease resistance genes in core eudicot including grapevine, indicating that R-genes typically show an unusually high turnover rate due to strong selection to keep up in a biological arms race with plant pathogens [31]. The presence of QTL region is usually screened with microsatellites markers, however, more than 900 total individuals from two additional progenies were required for the narrowing of this QTL suggesting that many genes outside the MrRUN1/MrRPV1 locus may frequently be also introgressed alongside this locus [32].

In order to address the introgression of additional genes and its consequences on gene expression in ripening berries, we conducted a comprehensive transcriptomic study on 102 single berries from non-resistant *V. vinifera* cultivars and resistant or non-resistant *V. rotundifolia* × *V. vinifera* derivative hybrids. Fruits were sampled across different stages of berry development. Following reads alignment on both *V. vinifera* PN400024.V4, *V. rotundifolia* var. ‘Carlos’ and ‘Trayshed’ genomes, Weighted Gene Co-expression Network Analysis (WGCNA), and Gene Ontology (GO) analysis allowed us to elucidate which LG12 region from *V. rotundifolia* was introgressed in the hybrids together with RUN1 and RPV1 genes. The multifaceted consequences of such introgression of *M. rotundifolia* genes on the transcriptome of the grapevine fruit are described.

## 2. Results

### 2.1. RNA-Seq Reads Statistics

The total number of reads per sample ranged from 35 to 45 million. Low-quality reads that were excluded from further analysis accounted for 11% of the total reads, reflecting the sequencing quality and preprocessing thresholds. Only 1.8% of high-quality reads did not align with the merged genomes. Among reads mapped on assigned features, 96% were mapped in single positions on the PN40024.v4 genome and 90% of these single-copy reads still mapped in a unique position on the merged genomes, indicating that they were genetically distant enough to warrant successful mapping to the right orthologs (Table S1).

### 2.2. Principal Component Analysis (PCA) Highlights the Development Program of the Single Berry

**Figure 1:**
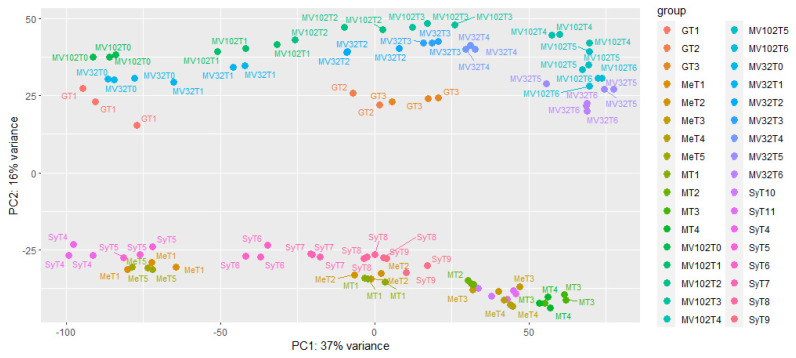
Principal component analysis of 102 single-berry RNA-seq samples, with gene expression monitored at various dates ranging from 3 to 10 depending on genotypes. PCA was performed on variance-stabilized transforms of RNA-Seq data on 29516 genes. G represents G5. Me and M represent two different Merlot clones, and Sy represents Syrah genotypes. MV32: G5 descendant devoid of the MrRUN1/MrRPV1 locus, MV102: G5 descendant introgressed for the MrRUN1/MrRPV1 locus. Within each cultivar, samples are ranked according to their respective sampling dates, indicating that the developmental stage has a major effect on the pattern of gene expression pattern than genotypes.

RNA expressions from 102 single berries were quantified following the alignment of pair-end reads to the merged *Vitis vinifera* ‘PN400024.v4’, *Muscadinia rotundifolia* ‘Trayshed’ and ‘Carlos’ genomes, plus the seven R genes available for the pertinent ‘52’ parent. A total of 29,516 genes were identified as expressed (to be considered as such, the sum of the normalized read count of a gene must be >1). A principal component analysis (PCA) was conducted following variance stabilizing transformation (vst) of HTSeq counts, with the first and second components accounting for 43% of the variance. As much as 37% of the variance of gene expression (PC1) is associated with berry development and ripening. Single berries inside triplicates were generally grouped at each developmental stage. Although *V. rotundifolia* descendants were clearly resolved from native *V. vinifera* cultivars on PC2, resistant and non-resistant varieties were not resolved in this PCA, indicating that the number of genes affected by MrRUN1/MrRPV1 introgression should be comparatively low (Figure 1).

### 2.3. Identification of Differentially Expressed Genes

**Figure 2:**
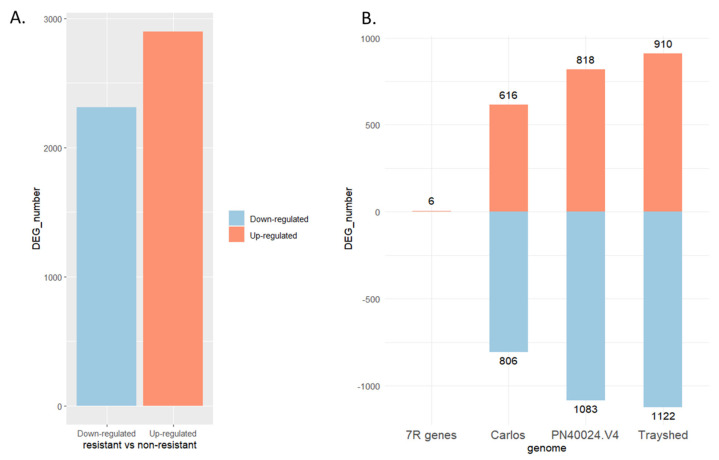
DEGs of berries with resistant versus non-resistant genotypes. (**A**) The *X*-axis represents down-regulated and up-regulated genes, while the *Y*-axis is the number of regulated genes. (**B**) The *X*-axis represents the reference genome, and 7R presents the seven resistance genes sequenced in the RUN1/RPV1 locus of *V. rotundifolia* 52 [32], which is the right Muscadinia genetic background of G5, MV102, and MV32 genotypes. Trayshed and Carlos stands for *V. rotundifolia* cv ‘Trayshed’, and ‘Carlos’ respective genomes. The *Y*-axis represents the DEGs number in each genome.

Transcripts expression values were compared between the resistant cultivars group (G5 and MV102 samples, including three and seven sampling dates, respectively) and the susceptible one (Syrah, Merlot clone1, Merlot clone2, and MV32 samples obtained at 8, 4, 5, or 7 dates respectively). Setting the log_2_ fold change threshold as 1, FDR < 0.05 then *p*-value < 0.02 yielded a total of 5211 differentially expressed genes (DEGs) among the 29516 expressed ones, including 2899 up-regulated and 2312 down-regulated ones (Figure 2A). Among the genes overexpressed in berries from resistant accessions, 832, 1003, 1058, and 6 genes were mapped on PN40024.V4 12X2, *V. rotundifolia* cv ‘Trayshed’ and cv ‘Carlos’ genomes plus seven *V. rotundifolia* cv ‘52’ RGA genes, respectively. Conversely, regarding down-regulated genes, respectively 937, 660, and 715 genes originated from the *V. vinifera* PN40024.V4 12X2, *V. rotundifolia* cv ‘Trayshed’ and ‘Carlos’ genomes (Figure 2B).

### 2.4. Functional Annotation of the Differentially Expressed Genes

**Figure 3:**
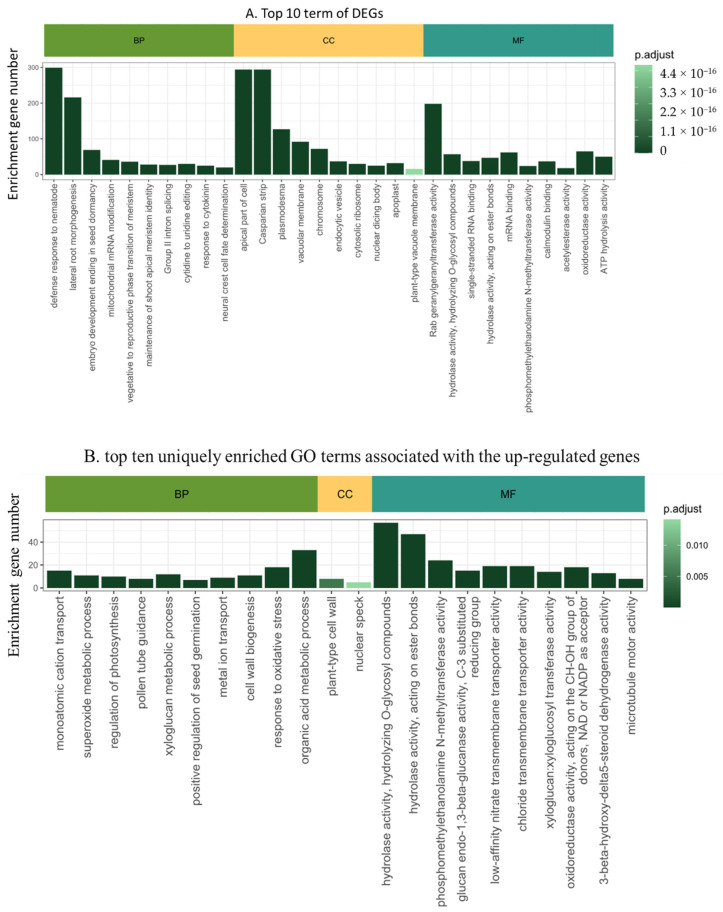

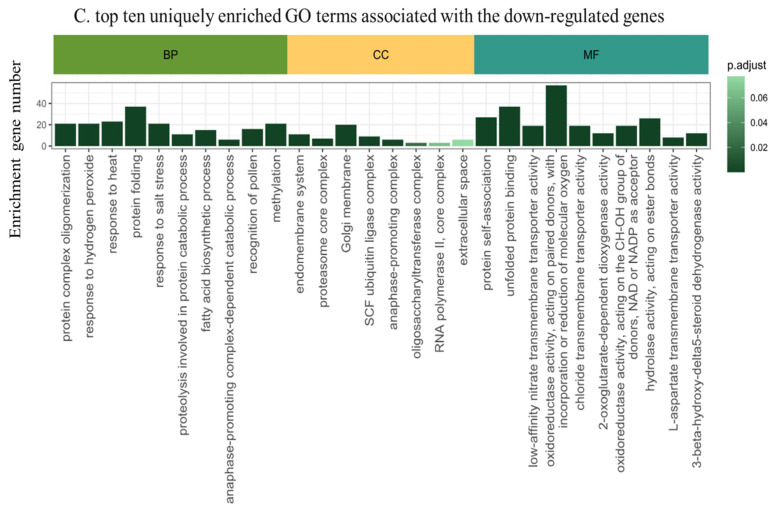
Gene ontology enrichment analysis of MrRUN1/MrRPV1 introgressed berries versus native *vinifera* ones. (**A**) Top ten GO terms of DEGs between non-resistant vs. resistant berries. (**B**) Top ten uniquely enriched GO terms in up-regulated genes. (**C**) Top ten uniquely enriched GO terms in down-regulated genes. CC, cellular component; MF, molecular function; BP, biological process. Gene Ontology enrichment plots show detected GO terms (under 0.05 in Fischer’s exact tests), color-coded by their adjusted *p*-value, and shifted in the *y*-axis depending on the number of genes matching this ontology.

We employed the DIANE bioinformatics package for the functional annotation of DEGs. The Gene Ontology (GO) enrichment analysis revealed an enrichment in 31 cellular components, 119 molecular functions, and 98 biological processes. In terms of biological processes (BP), the prominent enriched categories included defense responses to nematodes (GO:0002215), mitochondrial mRNA modification (GO:0080156), cell wall modification (GO:0042545), cytidine to uridine editing (GO:0016554), and responses to cytokinin (GO:0009735), etc. For cellular components (CC), the main categories were the apical part of the cell (GO:0045177), Casparian strip (GO:0048226), plasmodesma (GO:0009506), vacuolar membrane (GO:0005774), chromosomes (GO:0005694), etc. Lastly, the molecular functions (MF) identified were predominantly associated with Rab geranylgeranyltransferase activity (GO:0004663), G-protein beta/gamma-subunit complex binding (GO:0031683), monoatomic cation transmembrane transporter activity (GO:0008324), acyltransferase activity (GO:0016746), and chitin-binding (GO:0008061). The top 10 categorizations and their implications are further illustrated in Figure 3A. We compared the up-DEGs and down-DEGs GO terms in order to get the unique up- and down DEGs. The top 10 GO functional annotation terms were listed (Figure 3B,C). The up-DEGs unique GO term contains cell wall organization (GO:0071555), tRNA 5’-leader removal (GO:0001682), regulation of photosynthesis (GO:0010109), xyloglucan metabolic process (GO:0010411), response to oxidative stress (GO:0006979). On the other side, the down-DEGs unique GO term included SCF ubiquitin ligase complex (GO:0019005), response to cadmium ion (GO:0046686), oxidoreductase activity acting on single donors with incorporation of molecular oxygen, incorporation of two atoms of oxygen (GO:0016702), hydrolase activity, acting on esterbonds (GO:0016788), and fatty acid biosynthetic process (GO:0006633).

### 2.5. Weighted Gene Co-Expression Network Analysis (WGCNA)

**Figure 4:**
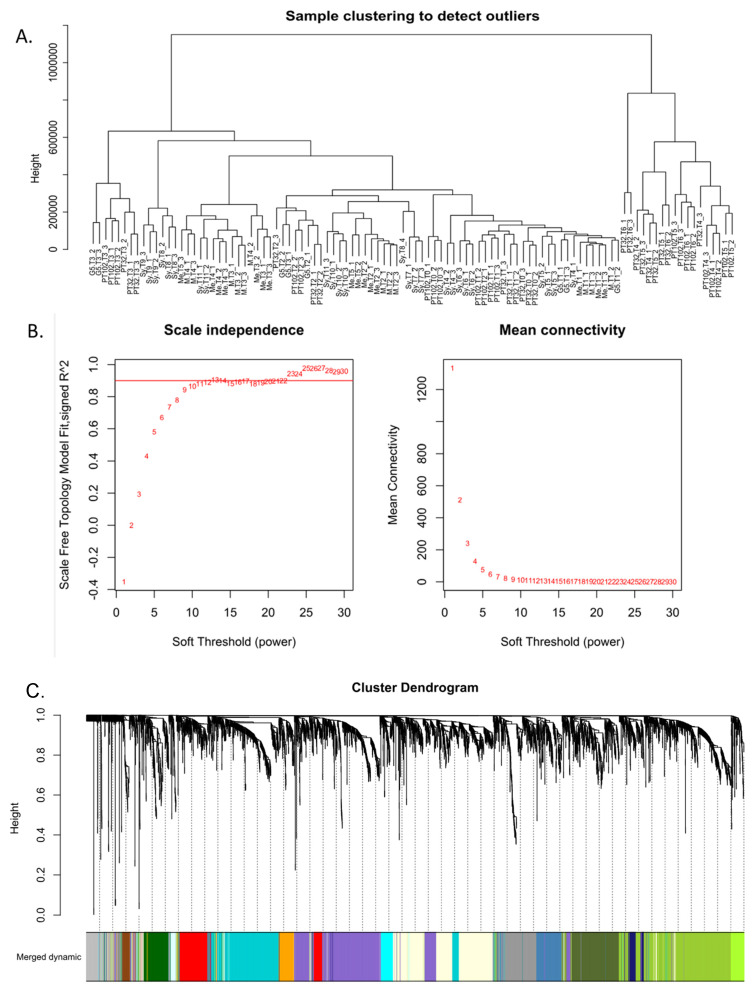
Construction of the gene co-expression network from DEGs in single berries from resistant vs. susceptible genotypes. (**A**) No glaring outlier emerged from sample clustering (**B**) Network topology analysis showed that at β = 10, the network satisfied the scale-free topology threshold of 0.9. When β = 10, network topology analysis showed that the mean connectedness was almost zero. (**C**) Gene dendrogram constructed by clustering dissimilarity (MEDissThres = 0.4). Color-coded modules represented by lines reflecting the consensus topological overlap. The cluster dendrogram at the top shows co-expressed genes. The branches and color bands at the bottom represent the assigned module. Every module has a distinct color that designates a group of co-expressed genes.

DEGs between berries from susceptible genotypes vs. those from MrRUN1/MrRPV1 introgressed ones were submitted to WGCNA. All samples were included in the WGCNA because the sample clustering dendrogram (Figure 4A) showed no glaring outliers. Next, before the gene co-expression network was built, the soft threshold power β was determined. The network satisfied the 0.9 scale-free topology threshold (Figure 4B), and with 10 soft-thresholding power, the mean connectedness was almost 0 (Figure 4B). Finally, 15 modules were identified using dynamic tree trimming and average hierarchical clustering (Figure 4C).

### 2.6. Identification of Key Modules Associated with Traits

**Figure 5:**
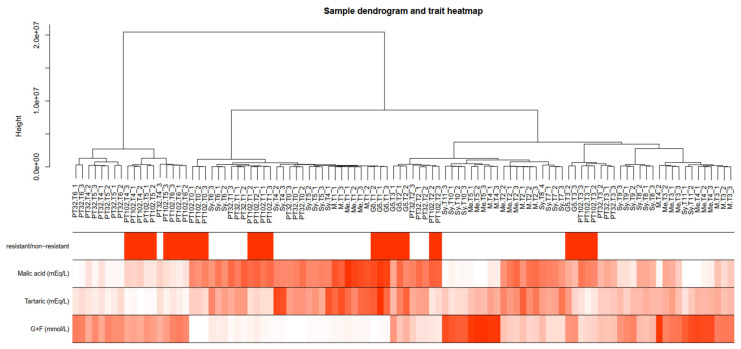
A dendrogram and trait heatmap of the samples. The leaves of the tree correspond to samples (method = “average”). The first color band underneath the tree documents the tolerant and susceptible status, in red and white, respectively. The remaining colored bands from top to bottom represent malic, tartaric, and sugar concentrations, respectively.

The dendrogram and heatmap analysis (Figure 5) revealed genetic and trait-based relationships among single berries, categorized into distinct groups by the presence/absence of the MrRUN1/MrRPV1 locus, and phenology markers like the concentrations of malic acid, tartaric acid, and glucose + fructose (Table S2). Sample clustering indicates four main branches, mostly associated with developmental stages, as shown by the relative concentrations of malic acid and sugars.

**Figure 6:**
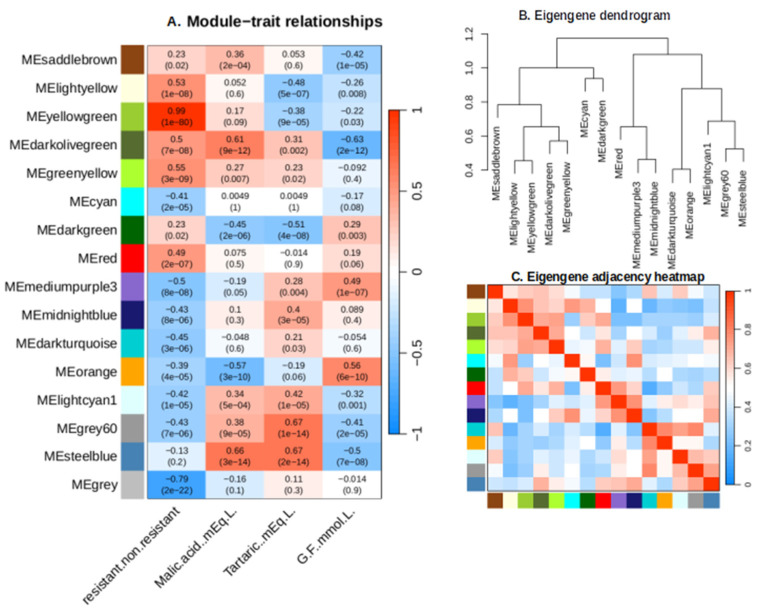
**A** module-trait associations diagram, Eigengene dendrogram, and Eigengene adjacency heatmap. (**A**) A heatmap showing associations between traits and gene expression modules. Figures indicate correlations coefficient and (*p* values). (**B**) A diagram showing the modules’ Eigengenes’ hierarchical clustering. (**C**) A heatmap showing the hub gene network’s adjacency relationships.

The trait/module relationships are presented in Figure 6. “ME steel-blue” and “ME dark olive green” modules exhibited strong positive correlations with malic acid concentration, and a strong negative one with sugar concentration, highlighting a possible role of this module gene in malic acid accumulation and green berry development, before being repressed during ripening (sugar accumulation phase). The “ME steel-blue” module displays positive correlations with tartaric acid concentration, indicating its transcripts might be involved in tartaric acid production or general dilution in the expanding berry. Notably, the yellow-green module exhibited the most robust positive correlation with the introgression of MrRUN1/MrRPV1 markers in berries (r = 0.99, *p* = 1 × 10^−80^), followed by the green-yellow module (r = 0.55, *p* = 3 × 10^−9^) (Figure 6). Gene numbers per module are presented in Figure S1. The green-yellow and yellow-green modules are found in the same branch of the Eigengene dendrogram and adjacency heatmap (Figure 6B,C). Consequently, both modules were considered for further analysis.

#### 2.6.1. Yellow-Green Module-Positively Associated with the Presence of MrRUN1/MrRPV1 Microsatellites Markers

**Figure 7:**
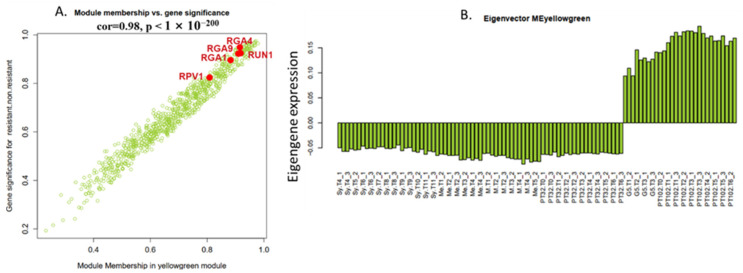
(**A**) Gene module membership (MM) vs. gene significance (GS) in the yellow-green module. MM represents the correlation between each gene expression profile and that of the module eigengene. GS represents the association between gene expression and resistance. GS and MM are exceptionally well correlated in this module (0.98, *p* < 1 × 10^−200^). Resistance gene analogs (RGA) sequences available at NCBI in the true parental MrRUN1/MrRPV1 locus [30] are indicated in red. (**B**) Eigengene expression pattern in 102 single berries.

**Figure 8:**
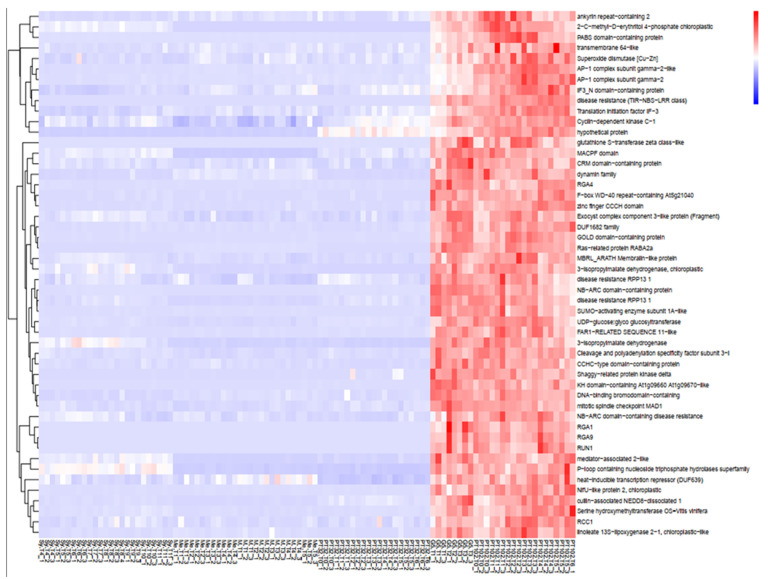
Heatmap of the expression levels of the top 40 annotated genes in the yellow-green module (Module Membership > 0.8) in different samples. Rows: single gene expression and function. Columns: susceptible and tolerant samples. Side Dendrogram: gene clustered according to their expression patterns (clustering_method = “complete”).

Gene Significance (GS) and module membership (MM) in the yellow-green module were found to be substantially associated (cor = 0.98, *p* < 1 × 10^−200^) by Pearson’s correlation analysis (Figure 7A). To eliminate module noise and keep only strongly co-expressed genes, we filtered genes with a module membership (MM) greater than the 0.8 threshold. Notably, the 5 R genes previously annotated in the MrRUN1/MrRPV1 locus [30] appeared within the 213 genes (0.8 < MM < −0.8) in the yellow-green module (Figure 7A). The yellow-green module Eigengene expression is specifically up-regulated in berries from the G5 and MV102 tolerant hybrids and down-regulated in the susceptible ones, including the MV32 sibling of MV102 (Figure 7B). Nineteen genes were negatively correlated with the Eigengene expression pattern in the yellow-green module (MM < −0.8), and 193 genes were positively correlated (MM > 0.8). According to the functional annotation of the top 40 genes in the yellow-green module heatmap (Figure 8), introgressed genes elicit a concerted molecular response associated with plant resistance, wherein the majority of genes within the module exhibit heightened expression levels in MrRUN1/MrRPV1 introgressed varieties. The up-regulated gene encompassed disease resistance (TIR-NBS-LRR class), NAC domain-containing protein, NB-ARC domain-containing disease resistance, LRR and NB-ARC domain disease resistance, heat-inducible transcription repressor (DUF639), transcriptional-regulating factor 1-like, auxin response factor 1, cell division topological specificity factor chloroplastic-like, a translation initiation factor of IF-3, and probable receptor kinase.

#### 2.6.2. Green-Yellow Module: G5 Specific Transcripts, Almost Lost in the Next Backcross

**Figure 9:**
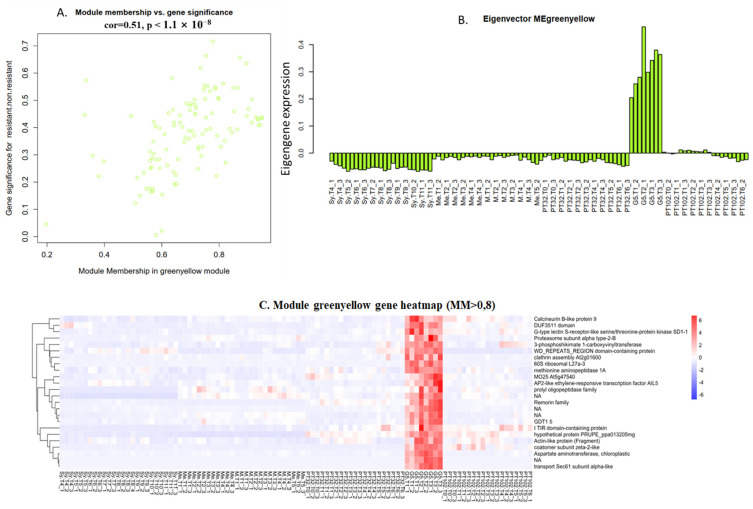
(**A**) A scatter plot of green-yellow module membership (MM) vs. gene significance (GS) (cor = 0.51, *p* < 1 × 10^−8^). MM represents the correlation between gene expression and that of module eigengene. GS represents the association between gene expression and treatment. (**B**) An Eigengene expression pattern. (**C**) A heatmap of the expression levels of green-yellow module genes across various samples (MM > 0.8). Rows: represent a single gene. Columns: represent different samples. clustering_method = “complete”.

**Figure 10:**
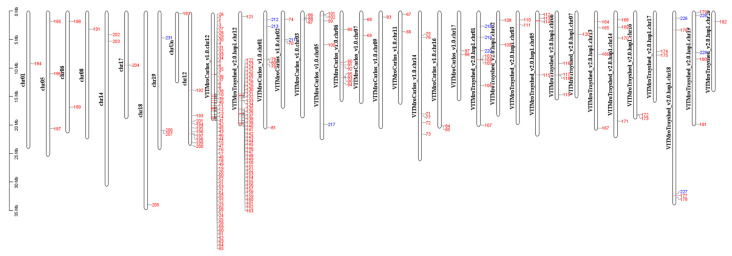
A representation of the genomic positions of yellow-green and green-yellow module genes (|MM| > 0.8). Red label: genes from the yellow-green module, blue label: genes from the green-yellow module, specifically expressed in G5.

Gene Significance (GS) and module membership (MM) were found to be substantially associated in the green-yellow module (cor = 0.51, *p* < 1 × 10^−8^) by Pearson’s correlation analysis (Figure 9A). 24 genes with |MM| > 0.8 are clustered in this module (Figure 9C). The expression of green-yellow module eigengene showed that this module is rather specifically expressed in G5 (Figure 9B), and almost lost in both MV102 and MV32. Annotated genes within the green-yellow module noticeably include AP2-like ethylene-responsive transcription factor AIL5, DUF3511 domain, G-type lectin S-receptor-like serine/threonine-protein kinase SD1-1, TIR domain-containing protein, etc. (Figure 9C). Most of these transcripts are preferentially mapped to the *V. rotundifolia* genomes (Figure 10).

### 2.7. Gene Introgressed with MrRUN1/MrRPV1 on Chromosome 12

G5 and MV102 share 193 overexpressed genes (MM > 0.8) in the yellow-green module. 117 genes among them, that is to say, 60% of the entire module, are located in chromosome 12 (65 genes in *V. rotundifolia* cv ‘Carlos’, 42 genes in *V. rotundifolia* cv ‘Trayshed’, and 10 genes in PN40024.V4) (Figure 10). G5 overexpressed genes also encompass green-yellow module genes (Figure 9), but these genes, which disappeared in MV102 and MV32, are not grouped in chromosome 12 (Figure 10).

**Figure 11:**
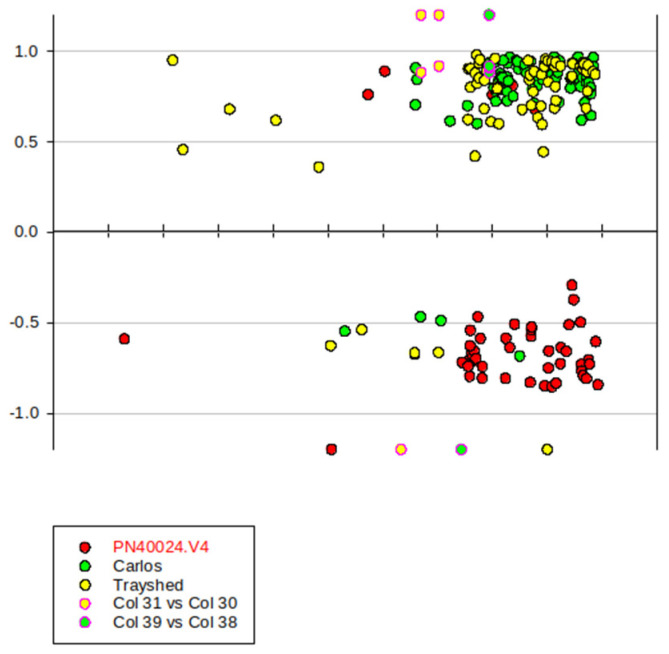
Genomic positions of yellow-green and green-yellow module genes associated with Run1/Rpv1 locus. X-axis: gene position in chr12. Y-axis: gene MM. The red point in the black circle represents the genes mapped to PN40024.V4; the green point in the black circle represents the genes identified in *V. rotundifolia* ‘Carlos’; the yellow point in the black circle represents the genes identified in *V. rotundifolia* ‘Trayshed’; Yellow point in the red circle represents the VMC4f3.1 microsatellite marker. The green point in the red circle represents the VMC8g9 marker.

The SSR markers VMC4f3.1 and VMC8g9 were located at chromosome 12 positions 11270828-11271019 and 17943471-17943607, in *V. rotundifolia* cv. ‘Trayshed’, and at 12811273-12811496 and 16731670-16731806 on *V. rotundifolia* cv. ‘Carlos’ markers, respectively [33]. The coordinates of VMC4f3.1 were used as a common reference, before checking the precise position of the genes overexpressed in resistant and susceptible genotypes, respectively (Figure 11). Remarkably, the genes that are overexpressed in MrRUN1/MrRPV1 introgressed genotypes are mostly located on the extremity of *Vitis rotundifolia* chromosome 12 that starts at the introgressed locus but extends well beyond the VMC4f3.1 marker. By contrast, the negatively correlated ones (i.e., those that are overexpressed in susceptible cultivars) are mapped to the orthologous LG12 part of *V. vinifera* PN40024.v4 genome.

## 3. Discussion

### 3.1. PCA and Gene Expression Profiles

The selection of single berries at duly characterized phenological stages, based on the evolution of sugar and acids during their developmental program, has proven to be a robust technique for investigating gene expression throughout the berry ripening process [34,35,36]. In the present work, a meta-analysis of single-berry RNA-seq data from different genotypes confirmed the clear clustering of triplicates in the PCA. PC1 highlights the variance due to development, while PC2 likely accounted for genetic differences between native *vinifera* and their *rotundifolia* hybrids. Sorting synchronized berries can avoid misinterpretation of phenological differences as genotypic differences. However, naive DEG analysis between introgressed and native genotypes was confused by unequal representativity of samples at specific developmental stages among genotypes. Supplied with a range of phenology-related quantitative traits, the WGCNA approach eliminated such confusion, and proved successful in the identification of *bona fide* genotype markers, as confirmed by the constitutive expression in healthy berries from introgressed genotypes of *V. rotundifolia* ‘52’ MrRUN1/MrRPV genes, together with other RGA in the same locus. Since the complete BAC sequences were absent from public repositories [37], and in order to check putative introgressions outside them, we attempted to identify the best orthologs of *V. rotundifolia* ‘52’ genes in other *V. rotundifolia* genomes. The reads were thus aligned against a merged reference grapevine genome comprising three genomes (PN40024.V4, *V. rotundifolia* cvs. ‘Trayshed’, and *V. rotundifolia* cv. ‘Carlos’ + 7 R resistance genes) providing comprehensive information for all varieties [38,39,40]. The BAM file was checked using IGV [41] to visualize the mapping of reads onto the merged genome. The analysis of random genes revealed no significant bias in the read alignment process (Figure S2).

### 3.2. Merged V. vinifera and V. rotundifolia Genome

Due to its seniority, comprehensive sequencing, and well-documented structure, the highly homozygous *Vitis vinifera* genotype PN40024.V4 (https://grapedia.org/genomes/ accessed on 22 July 2024) provides a foundational framework for grapevine studies, particularly in gene expression and functional genomics [42]. Many RNA-seq studies have used this genome as a reference, sometimes when dealing with *non vinifera* species [13,14,18,35]. Selecting the appropriate reference genome is critical for RNAseq analysis, particularly with genetically distant genotypes such as deriving from *V. rotundifolia*, previously considered as a distinct, non-interfertile genus (*Muscadinia rotundifolia*). When analyzing viral pathogens, some researchers align RNA-seq data to a combined grapevine-virus reference genome [43].

The obvious limitations of a single reference genome can be overcome by de novo assembly of reference transcriptomes tailored to specific organisms [17,44]. This approach is advantageous in non-model organisms or highly heterozygous varieties, revealing the genomic complexities of different grapevine cultivars or hybrids [45,46].

Repetitive structure of the TIR-NBS-LRR family (Toll/Interleukin-1 Receptor, Nucleotide-Binding Site, Leucine-Rich Repeat) [47]. Massonnet et al. compared NLR genes across different haplotypes of *V. rotundifolia* (‘Trayshed’), particularly the Run1.2 and Run2.2 loci. The clustering of RUN1/RPV1 TIR-NBS-LRRs proteins with Run1.2 ones, revealed an allelic link between Run1.2 and RUN1/RPV1, characterized by the association of two TIR-NBS-LRRs from the Run1.2 haplotypes with MrRPV1 from *V. rotundifolia* G52, although differing in the number of motifs in their LRR domain revealed. Furthermore, variations in LRR domains imply that these TIR-NBS-LRRs may be particular to various infections and/or effectors [48].

In our RNA-seq analysis, we employed the recent *V. rotundifolia* ‘Carlos’ and *V. rotundifolia* ‘Trayshed’ genomes to improve the interpretation of RNAseq data on *Vinifera x rotundifolia* hybrids derived from *M. rotundifolia* ‘G52’ [7,49]. However, extensive genetic studies on both wild and cultivated varieties highlighted the inherent genetic diversity within *V. rotundifolia* species [50,51]. Significant genetic variations may exist between the sequenced ‘Carlos’ or ‘Trayshed’ genomes and *M. rotundifolia* ‘G52’, as the parent of the *Muscadinia* derivative hybrids used in this study, and a ubiquitous MrRUN1/RPV1 provider in European breeding programs. These variations could blur gene expression profiling. Therefore, our findings should be considered within the context of these potential genomic discrepancies. It is encouraging in this respect that 85% of RNAseq reads specifically mapped to one single position in the merged genotypes, either in *V. vinifera*, Feechan G52 RGAs [32], *V. rotundifolia cv.*‘Trayshed’ or ‘Carlos’.

### 3.3. Regulation of Genes Related to Pathogen Response

The plant immune system utilizes two main mechanisms for pathogen perception: pattern-triggered immunity (PTI) and effector-triggered immunity (ETI) [52]. PTI involves membrane-bound pattern recognition receptors (PRRs) that detect pathogen-derived molecules externally, while ETI is activated by intracellular receptors that recognize specific pathogen effectors inside the cell. This often leads to a hypersensitive response involving localized cell death [52]. Both PTI and ETI mechanisms-related transcripts were found constitutively expressed in healthy berries introgressed with the MrRUN1/RPV1 locus. The intracellular receptors mainly consist of nucleotide-binding domain NLR proteins, which can include either Toll/interleukin-1 receptor/resistance (TIR) or coiled-coil (CC) domains. Structural studies show that upon effector recognition, these last proteins undergo structural changes that enable them to form oligomeric complexes targeted to the plasma membrane, which initiate cell death signaling via calcium channel activity. TIR domain-containing proteins from several bacterial and one archaeal species can remove the nicotinamide moiety from NAD-capped RNAs (NAD-RNAs) [53].

In the present study, in addition to the 6 RGA, 15 genes annotated as TIR, 12 genes annotated as CC-NBS-LRR, 46 genes as LRR receptors, and 8 genes belonging to the CC-NBS-LRR class were found among the DEGs (Table S3). This finding highlights the critical role of oxidoreductase and NAD+ nucleosidase activities, essential for redox reactions and NAD+ metabolism, in enhancing disease resilience in berries [54,55,56]. In Qu’s study, TIR domains from grapevine TIR-NLRs (RPV1) were shown to induce cell death [57]. Further downstream, TIR domains of EDS1 proteins form complexes with PAD4 and SAG101, which interact with NRG1 and ADR1 to propagate immune responses like cell death [58]. Overall, recent research suggests that TIR or additional domains act as integrated decoys recognizing effectors from pathogens. Proteins homologous to integrated decoys are suspected to be effector targets and involved in disease or resistance. A multilayered regulation of pathogen receptors multimerization driven by interactions among nucleotide-binding domains may act as a signal to activate the grapevine immune system. ZBED proteins, containing a decoy BED domain, regulate rice’s defense against the blast fungus *Magnaporthe oryzae* [59]. Ma’s study revealed that the plant TNL receptor RPP1 recognizes the pathogen effector ATR1 via C-JID and LRR domains, triggering tetramer formation that activates NAD+ hydrolysis and subsequent cell death [60]. Multiple organelles are involved in defense response, including chloroplasts and peroxisomes for hormone production as well as the nucleus, endoplasmic reticulum, and Golgi apparatus for antimicrobial protein production. A pair of LRR kinase-like disease resistance genes orthologs regulates rice response to increased temperature [61].

Defense genes triggered by *P. viticola* infection in MrRPV1 transgenic *V. vinifera* Syrah leaves were compared with those constitutively expressed in berries from MrRUN1/RPV1 introgressed genotypes [57] (Table S4). The Vitvi03g00882 gene showed a 2.3-fold increase in expression in berries from fungi-tolerant genotypes when compared to susceptible ones. This gene is paralogous to the two Wall-Associated Kinases (WAKs) triggered by *Plasmopora viticola* infection in RPV1 transgenic Syrah, from 18 to 36 hours post-inoculation [57]. WAKs are known to trigger the plant’s innate immune response by acting as receptors for cell wall-associated oligogalacturonides [62]. Wall-associated receptor kinase-like and G-type lectin S-receptor-like proteins contribute to resistance by interacting with leucine-rich repeat (LRR) domains [63,64]. In the present study, 22 G-type lectin S-receptor-like proteins exhibited differential expression between susceptible and fungi-tolerant genotypes. Of these, half showed up-regulation, while the other half demonstrated down-regulation in fungi-tolerant genotypes (Table S3). One up-regulated G-type lectin S-receptor-like protein (Vitvi13g02551/VIT_13s0156g00590) aligns with the one up-regulated in MrRPV1-transgenic *V. vinifera* leaves following 36 h infection with *P. viticola*. In contrast, three down-regulated G-type lectin S-receptor-like proteins (Vitvi13g02552/VIT_13s0156g00580, Vitvi13g02553/VIT_13s0156g00550, Vitvi04g02226/VIT_04s0044g00680) in our study differ from the prior study, which reported an increase at 36 hpi in MrRPV1-transgenic plants (Table S4). 4 DUF domain-containing genes (VITMroCarlos_v1.3.g11564, VITMroTrayshed_v2.0.hap1.chr12.ver2.0.g155730, VITMroTrayshed_v2.0.hap1.chr12.ver2.0.g155780, VITMroTrayshed_v2.0.hap1.chr12.ver2.0.g157440) are annotated in *the* yellow-green module. The DUF642 gene from the Chinese grape species *V. quinquangularis* accession Danfeng-2 encodes a cell wall protein involved in both berry growth and defense responses to *Erysiphe necator* and *Botrytis cinerea* [63]. Additionally, Vitvi16g01485, the homolog of VIT_10s0042g00930 annotated as stilbene synthase, is downregulated in berries from fungi-tolerant genotypes, which contrasts with the induction of stilbene synthesis in grapevine leaves from resistant cultivars triggered with the pathogen [57,65]. VviWRKY10 and VviWRKY30 have also been shown to play crucial roles in grapevine leaves defense against powdery mildew. VviWRKY10 acts as a negative regulator of salicylic acid (SA)-dependent defense by binding to the W-boxes in the promoters of SA-related genes and inhibiting their transcription. Conversely, VviWRKY30 promotes ethylene (ET)-dependent defense by binding to W-boxes in the promoters of ET-related genes and enhancing their transcription. Additionally, these transcription factors can mutually inhibit each other’s expression, ensuring a balanced defense response [66]. In our study, we identified DEGs with WRKY domains and W-box motifs, suggesting further layers of complexity in the regulatory networks involving WRKY transcription factors.

No matter the above, the present results on healthy vines, in the absence of contaminations in their environment, clearly show that the large introgression of *muscadinia* genes outside the RUN1/RPV1 locus triggered huge constitutive transcriptomic changes when compared to RPV1 single gene transgenic Syrah briefly post-inoculation. The present results show that newly bred pathogen-resistant grapevine genotypes may include and constitutively express substantially more genes than believed based on microsatellite markers. We can anticipate that the introgression should become dramatically complex when pyramiding resistance genes from different wild species [28,67].

## 4. Materials and Methods

### 4.1. Grapevine Genotypes

**Figure 12:**
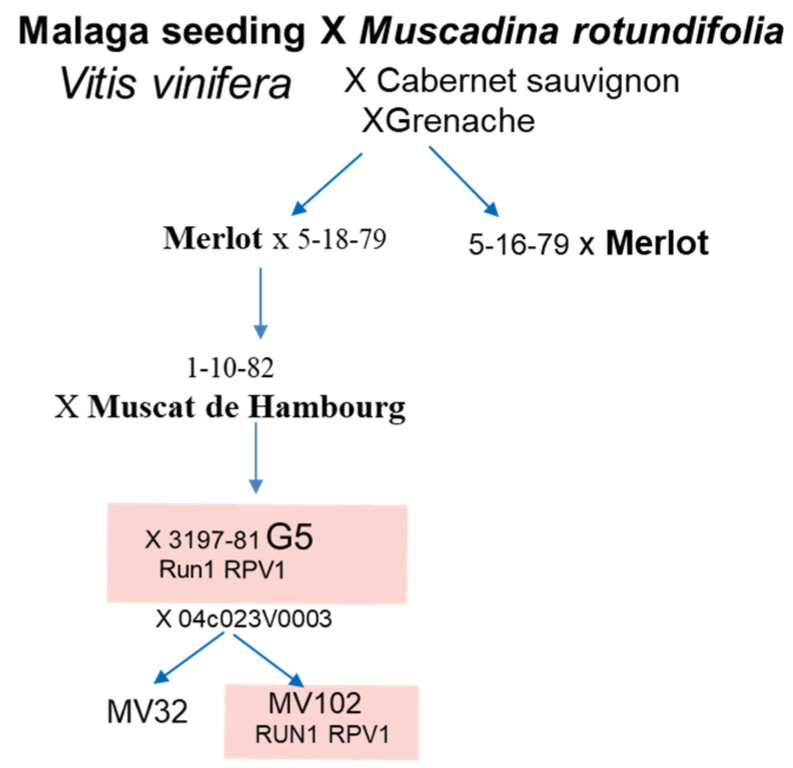
Pedigree of the fungus-tolerant genotypes (adapted from Ojeda et al., 2017 [70]. G5 is a macrovine phenotype and MV102 and MV32 are two microvine lines, the first one carrying the RUN1/RPV1 locus and the second without it.

The study encompassed five grapevine genotypes, including the disease-resistant G5 and MV102 hybrids, and the susceptible cultivars *V. vinifera* Syrah, Merlot, and MV32 hybrid. To create a molecular scale of grapevine berry development, we relied on the RNA-sequencing dataset consisting of 102 samples, part of which was published by [35,68]. Samples were collected from before véraison to ripening every 7–10 days in different locations (Table 1). The disease-resistant G5 hybrid, which displays the sugarless trait [69], results from four pseudo-backcrosses of the *V. vinifera* × *M. rotundifolia* NC6-15 F1 hybrid with *V. vinifera* cultivars [7]. G5, also named 3197-81B, was cultivated in the INRAE experimental unit of Pech Rouge, France (43.14° North | 3.14° East). Notably, G5 carries the MrRUN1/RPV1 locus, known for enhancing tolerance to fungal infections [32]. The two hermaphroditic semi-dwarf microvines [65], designated MV32 and MV102 [35], derive from a fifth backcross between the 04c023V0003 female microvine [49] and the G5. The MV102 microvine possesses the MrRUN1/RPV1 locus, contributing to enhanced tolerance to *Erysiphe necator* and *Plasmopara viticola*, two major specific fungus diseases in grapevine, while the susceptible MV32 lacks this locus (Figure 12). The two-year-old potted microvines were cultivated in a semi-controlled greenhouse with a temperature range of 25 °C during the day and 15 °C at night. The greenhouse maintained a vapor pressure deficit of about 1 kPa and a photoperiod of 12 h of light per day. Syrah and Merlot samples are described, respectively, in [35,68] (Table 1).

### 4.2. Single Berry Sampling

For sampling, only healthy undamaged bunches were considered for analysis. To avoid circadian cycle influences, berries were sampled at the same time of the day, between 9 AM and 11 AM. Berries were rapidly deseeded and wrapped in tin foil, then frozen in liquid N2, and stored at −80 °C. Single berries were cryogenic ground with a mortar and a pestle to a fine powder under liquid N2 manually. Around 100 mg of frozen powder was used for HPLC analysis of soluble sugar and major organic acids to form biological replicates with nearly similar primary metabolite contents and developmental stages. The remaining powder was cryopreserved in liquid nitrogen for subsequent RNA extraction (Table 1).

### 4.3. HPLC Analysis of Primary Metabolites in Single Berries

One hundred mg of frozen powder sample was subjected to a 6× dilution with a 0.25 N HCl solution. After thorough shaking, the mixture was allowed to stand overnight at room temperature. Subsequently, the samples underwent centrifugation at 13,000× *g* for 10 min, and a supernatant aliquot was further diluted 10× using a solution of 5 mM H_2_SO_4_ containing 600 µM acetic acid as an internal standard. The prepared samples were then transferred to high-performance liquid chromatography (HPLC) vials for glucose, fructose, malate, and tartrate determination, as in Rienth et al. [13].

### 4.4. RNA Extraction and Sequencing

For every developmental stage, triplicate samples of single berries were chosen, guided by considerations of relative growth, sugars, and organic acids. An individual RNA extraction and subsequent library preparation was conducted as in [13]. Sequencing was performed on an NGC Illumina HiSeq3000 in paired-end mode with 2 × 150 bp reads, at the Genotoul platform of INRAe-Toulouse. A total of 102 samples were obtained, including those of fungus-tolerant G5 and MV102 hybrids, and the susceptible cultivars *V. vinifera* Syrah [35], Merlot [68], and MV32 hybrid samples.

### 4.5. Methodology for Transcriptome Analysis

After preprocessing raw reads with fastp (version 0.20.1) to eliminate adaptor sequences and discard low-quality or empty sequences (using the following parameters: -q 30 -u 40 -l 36 --cut_tail --cut_tail_window_size 3 --cut_tail_mean_quality 30 --detec t_adapter_for_pe), the resulting high-quality reads were aligned to a merged reference grapevine genome comprising three genomes (*V. vinifera* PN40024.v4 reference genome, *V. rotundifolia* cv ‘Trayshed’ and cv ‘Carlos’, and the few resistance genes from the original *V. rotundifolia* cv ‘52’ parent available at NCBI) using Hisat2 (version 2.2.1) with default parameters. This yielded an average of 59.221.274 mapped reads per sample (σ = 14.985.062). Subsequently, aligned reads were counted using HTSeq-count (version 0.13.5) using the following options: -mode=union --order=pos --nonunique al -t mRNA -t Parent -s reverse, with the merged annotation between PN40024.V4 (https://integrape.eu/resources/genes-genomes/genome-accessions/ accessed on 22 July 2024), *Vitis rotundifolia* cv. ‘Carlos’ (https://zenodo.org/record/7944875 accessed on 22 July 2024) and cv ‘Trayshed’ (https://grapegenomics.com/pages/Mrot/download.php accessed on 22 July 2024). DEGs from resistant cultivars and non-resistant cultivars were detected using DIANE [71]. The raw count data were first normalized using the TMM methodology, and then low-count genes were removed. Differential expression analysis was then performed through DIANE with an FDR < 0.05 then *p* value < 0.02 and a log2 fold change cutoff of 1.

### 4.6. GO Annotation Analysis

When analyzing DEGs from genomic data, it is crucial to understand the biological context of these genes. The DIANE tool, when integrated with the clusterProfiler R package, provides a robust framework for this analysis. ClusterProfiler uses Fisher’s exact test based on a hypergeometric distribution to statistically evaluate which Gene Ontology (GO) terms are overrepresented among DEGs. Using R version 4.1.2 (1 November 2021), we visualized the bar plot of enriched GO terms with associated gene counts and *p*-values.

### 4.7. WGCNA Analysis

The WGCNA package in R software was utilized to construct a co-expression network [66] for the identification of related gene modules and eigengene to traits of interest. According to Pearson’s correlation matrices, we constructed a weighted adjacency matrix. To emphasize the weak correlations and strong correlations between genes, Module Eigengenes were correlated with sample traits, and corresponding *p*-values were calculated. For coding for the traits see Table S2. For each trait, driver genes within a module were determined based on transcripts with the highest absolute gene significance and module membership, as calculated by WGCNA [72]. Gene significance was evaluated by correlating a transcript’s expression profile with the sample trait, while module membership scores were determined by assessing the correlation between a module eigengene and the expression profile of each transcript. All reported *p*-values were directly extracted from the WGCNA output. The total connectivity and intramodular connectivity were calculated with weighted Pearson correlation functions.

## 5. Conclusions

RNA-seq fragments were aligned to a composite genome integrating the *Vitis vinifera* reference genome, several *Muscadine* genomes, and the RGA inside the homologous MrRUN1/MrRPV1 locus. A continuum of transcripts overexpressed in fungus-tolerant genotypes aligns with the distal 4–5 Mb region of *V. rotundifolia* chromosome 12, starting with the MrRUN1/MrRPV1 locus, while those preferentially expressed in susceptible varieties align with the orthologous *V. vinifera* region. This demonstrates that this distal end of chromosome 12 remained recalcitrant to internal recombination during successive backcrosses with *V. vinifera*. Some *V. rotundifolia* genes expressed in fungus-tolerant genotypes were also observed outside this region, though to a lesser extent. Overexpressed genes in developing berries, either introgressed from *V. rotundifolia*, or regulated by them in the *V. vinifera* genome, span various functional groups, particularly calcium signaling, hormone signaling, transcription factors, plant-pathogen interactions, disease resistance proteins, ROS detoxification, and phenylpropanoid biosynthesis.

## Figures and Tables

**Table 1:** RNA-seq sample.

Genotype	Traits	Sample Year	Location	Dates of Sampling	Sample Number
G5	resistant	2021	Pech Rouge	3 dates	8
MV102	resistant	2018	Greenhouse	7 dates	21
MV32	non-resistant	2018	Greenhouse	7 dates	21
Syrah	non-resistant	2018/2019	SupAgro campus	11 dates	25
Merlot clone1	non-resistant	2022	Bordeaux	4 dates	12
Merlot clone2	non-resistant	2022	Bordeaux	5 dates	15

## Data Availability

All Syrah raw transcriptomics reads have been deposited in the NCBI Sequence Read Archive (http://www.ncbi.nlm.nih.gov/sra accessed on 22 July 2024) with the BioProject ID PRJNA862686. All G5 raw transcriptomics reads have been deposited in the NCBI Sequence Read Archive with the BioProject ID PRJNA1118503. All MV102 and MV32 raw transcriptomics reads have been deposited in the NCBI Sequence Read Archive with the BioProject ID PRJNA1121615.

## Supplementary Materials

The following supporting information can be downloaded at https://www.mdpi.com/article/10.3390/plants13152095/s1: Figure S1: Module gene number; Figure S2: Reads from the different genotypes selectively mapped to the MrRUN1 gene; Table S1: RNA-seq mapped merged genome reads; Table S2: Single berry traits; Table S3: DEGs annotation; Table S4: Qu’s paper gene blast result.
