# Supplementary material for: Vitis rotundifolia Genes Introgressed with RUN1 and RPV1: Poor Recombination and Impact on V. vinifera Berry Transcriptome
Source: Plants (Basel). 2024 Jul 29;13(15):2095. doi: 10.3390/plants13152095 (PMC11314213; doi:10.3390/plants13152095)
Supplement: Supplementary file 1 [file plants-13-02095-s001.zip › Supplementary Figure.pdf]

Supplementary Materials:

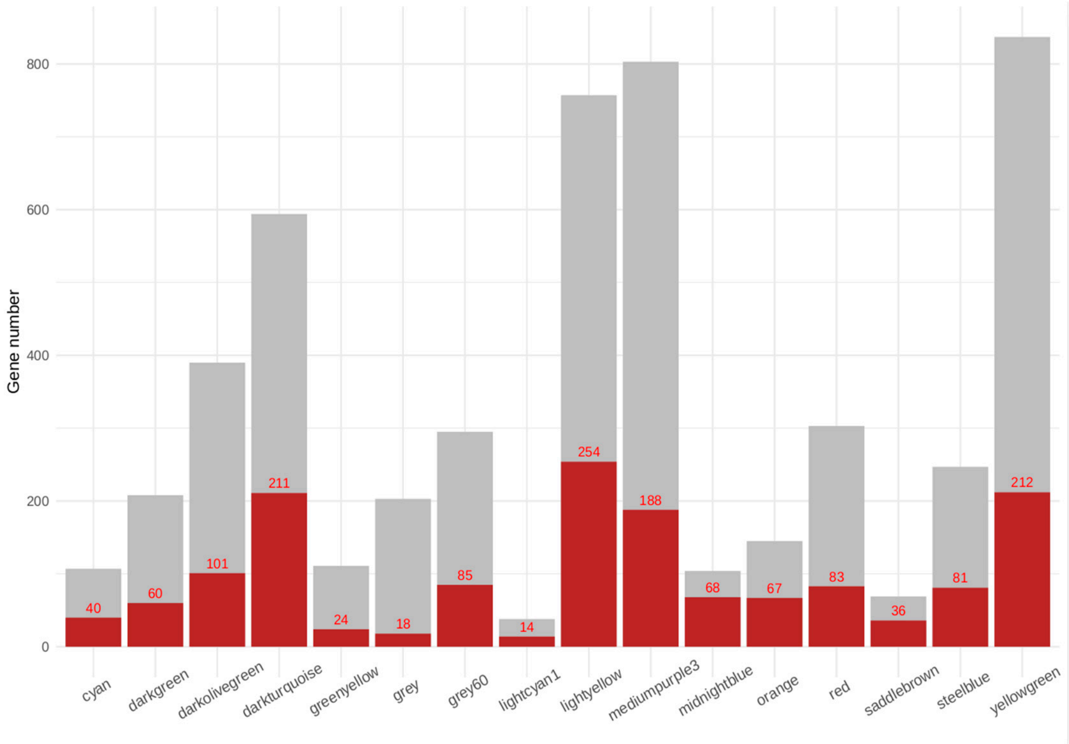

Figure S1. Module gene number. Grey: Raw module gene number, Red: filtered |MM|>0.8 module gene number.

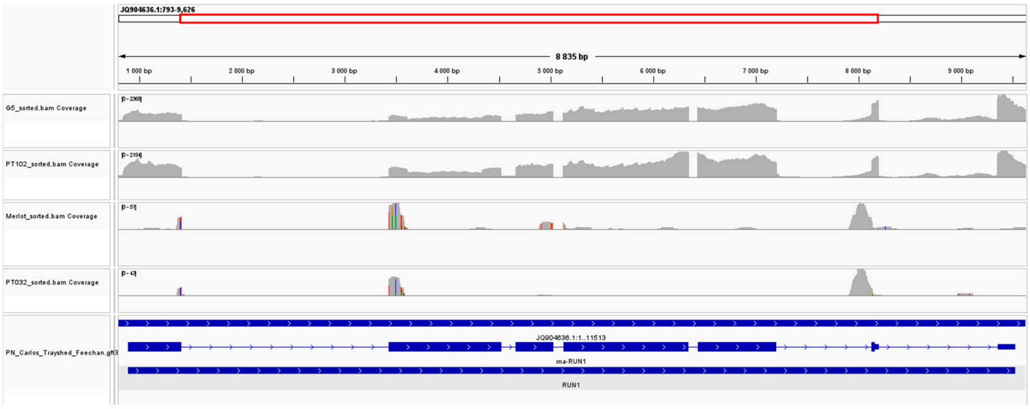

Figure S2. Reads from the different genotypes selectively mapped to the MrRUN1 gene.
